# Circumstances and Structure of Occupational Sharp Injuries among Healthcare Workers of a Selected Hospital in Central Poland

**DOI:** 10.3390/ijerph15081722

**Published:** 2018-08-10

**Authors:** Anna Garus-Pakowska, Magdalena Ulrichs, Ewelina Gaszyńska

**Affiliations:** 1 Department of Hygiene and Health Promotion, Medical University of Lodz, 90-647 Lodz, Poland; ewelina.gaszynska@umed.lodz.pl; 2 Department of Econometrics, University of Lodz, 90-214 Lodz, Poland; magdalena.ulrichs@uni.lodz.pl

**Keywords:** occupational exposures, needlestick/sharp injuries, healthcare workers, prevalence, injury rates, postexposure prophylaxis, reporting, surveillance, legal regulations

## Abstract

(1) Background: An analysis of work-related sharp injuries in Healthcare Workers (HCWs) based at a selected hospital in Central Poland by presenting the frequency of accidents, injury rates, and identifying circumstances of Needle Sticks and Sharp Injuries (NSSI) and giving Post-Exposure Procedures (PEP). (2) Methods: A retrospective analysis of medical documentation regarding work-related NSSI at a district hospital located in central Poland; over the period 2010–2017. The study group included HCWs who had an accident while on duty. (3) Results: Most injuries were reported by nurses and staff over 40, on the morning shift. The most common injuries were using a needle. The most exposed part of the body were fingers. The average annual injury rates were: 1.22/100 Nurses; 2.02/100 doctors; 1.34/100 hospital beds; and 8.59/100,000 inpatient days. The rates for 3-year periods, after the implementation of legal regulations were higher than before. Conclusion: Injuries rates are more reliable for comparison than frequency. Legislation on the need to register injuries seems to be necessary. In the supervision of work safety of personnel, reliable reporting of all injuries by the respective HCWs plays a key role.

## 1. Introduction

Sharp injury is defined as the parenteral introduction into the body of blood or other potentially infectious material by a hollow-bore needle or sharp instrument, including, but not limited to, needles, lancets, scalpels, and contaminated broken glass used during the performance of duties [1]. The main risk from a sharp injury is the potential exposure to infections such as blood-borne viruses (BBV). This can occur where the injury involves a sharp instrument that is contaminated with blood or a bodily fluid from a patient. The blood-borne viruses (BBV) of most concern include Hepatitis B (HBV), Hepatitis C (HCV), and Human Immunodeficiency Virus (HIV). The World Health Organization assigns a significant role to injections performed using contaminated equipment, or carried out inappropriately (i.e., unused or unchanged gloves when injecting other patients)—as procedures that carry a high risk of HBV and HCV infection [2]. The risk of infection of exposed persons (with a single contamination from a contaminated needle) is determined at 10–30% for HBV [3] and between 1.8–10% for HCV [4]. It is definitely higher than the risk of HIV infection, which for a single percutaneous contact (puncture) is assumed at 0.3%, and when exposed to mucous membranes it is 0.1% [5]. In 2000, contaminated syringes around the world caused 21.7 million cases of HBV infection, 2 million cases of HCV and 260,000 cases of HIV infection [5,6]. Given the nature of the work, viral blood-borne infections are considered occupational diseases among health care workers (HCWs). In Poland, diseases with infective etiology are responsible for over 60% of all occupational diseases among medical staff [7].

A register of potential occupational exposures should be kept in every healthcare institution, and every hospital employee (not only medical) should know the procedures to follow after contact with potentially infectious material. Every case of such exposure should be reported to the physician responsible for the PEP or to the infection control team, whose task is to assess the possibility of blood-borne infection and to adopt further proceedings depending on the type of exposure. Unfortunately, it is estimated that most of the events of exposure to the infectious agent are not documented in any way (i.e., reported to superiors or persons entitled to do so) [8,9,10,11,12]. In the case of percutaneous exposure, the determination of occupational risk is related to the type of activities performed that pose, or do not pose, such a threat. Therefore, it is important to assess whether an employee has regular contact with blood or other potentially infectious body fluids, whether there is a risk of injury with a contaminated needle or sharp tool, and the frequency of the above risky situations.

The main aim of this study was to analyze the data on occupational exposures among employees of a selected powiat hospital in the Lodzkie Voivodship, over the period 2010–2017, based on a register of needlesticks and sharp injuries (NSSI). The detailed aims included: (1) Analysis of the occurrence of injuries in particular years, taking into account the frequency of exposure, its circumstances, location of wounds, professional groups of employees, and the place of providing services; (2) analysis of the injuries rates; and (3) determining the frequency of post-exposure proceedings (PEP). The practical goal was to answer the question of whether legislation requiring mandatory reporting of NSSI brings real results.

## 2. Materials and Methods

The study bears information on work-related sharp injuries which happened to medical personnel, in the period 2010–2017, employed at a district hospital in Central Poland. The study group included HCWs, who had an accident while on duty. Data on the needlesticks and sharp injuries were obtained from unpublished hospital records. The authors used the register of sharp injuries, based on the exposure card in which the following data were recorded: Date and time of the incident, name and surname of the employee, organizational unit of the hospital in which the employee was employed, employee’s profession, place of injury, circumstances of wounding (during which action did the wound took place), and the type of tool that wounded the employee. In addition, the implementation of PEP is noted. Inside the hospital, the recording of injuries and wounds is carried out by a worker for Occupational Safety and Health. The analysis covered all recorded cases of occupational exposure to potentially infectious material. The hospital has a procedure to follow, after exposure to blood and other potentially infectious material, which determines how the employee should act immediately after the exposure. This procedure applies to all medical personnel exposed to HIV, HCV, and HBV. They include: Nurses, doctors, paramedics, midwives, and ward maids. The aim of the post-exposure procedure, is to determine the rules of conduct of the employee in case of exposure to infectious material. In the Łódź Province an infectious ward is designated as a consultative center. Every hospital employee who has been occupationally exposed is provided with 24-h medical consultation, seven days a week.

For the purpose of the statistical analysis, the authors used the Statistica 12.0 and Stata 15 programmes. They applied elements of a descriptive analysis for the evaluation of the injury rates in medical personnel. Sharp injuries were evaluated by: profession, medical tool, intervention, place of injury, location of wounds, kind of organizational unit, type of department, age of the employee (20–30 years, 31–40 years, 41–50 years, 51–60 years, >60 years), and time of day of the accident (duty hours—in the morning, in the afternoon, in the evening). For the purpose of evaluating a correlation between variables, the chi-square Pearson independence test was applied. All tests were verified at a 5% significance level. To determine the degree of correlation between the measured variables, the Pearson’s contingency coefficient was calculated. Injury rates to personnel who were involved in subsequent years were also calculated according to the following formulas:

*Injury rate depending on the profession*

$$IR_{p}=\frac{n_{1}}{N_{1}}100\;$$

where,

*n*_1_—number of injuries in individual groups of workers;

*N*_1_—number of workers at risk of injuries.

Due to various forms of employing workers (i.e., contracts of employment, orders, external companies providing cleaning services), the only comparable data concerned two professional groups: Nurses and doctors. For this reason, we have also calculated the injury rate per 100 hospital beds (*IR_b_*):

*Injury rate depending on the number of hospital beds*

$$IR_{b}=\frac{n_{2}}{N_{2}}100\;$$

where,

*n*_2_—number of injuries;

*N*_2_—number of hospital beds.

Only those departments affected by injury (the number of hospital beds) were considered for the calculation of *IR_b_*.

The most precise way to estimate the incidence in epidemiology is calculating the coefficient, considering one-day ones. [13]. For this reason, we also calculated the injury rate considering the number of so-called inpatient-days (*IR_c_*). One inpatient-day, is a unit of computation equal to one day of stay in hospital by one patient.

*Injury rate depending on the number of inpatient-days*

$$IR_{c}=\frac{n_{3}}{N_{3}}100,000\;$$

where,

*n*_3_—number of injuries = *n*_2_;

*N*_3_—number of inpatient-days.

Only those departments affected by injury (the number of inpatient-days) were considered for the calculation of *IR_c_*.

The director of the hospital and the director for medical affairs granted permission to access the medical records.

## 3. Results

### 3.1. Characteristics of the Hospital and the Study Group

In the years 2010–2016, there were 12 departments in the structure of the examined hospital with an average of 321 beds for internal diseases, internal diseases with cardiological profile, general surgery, obstetrics with gynecology, pediatric surgery, pediatrics, pulmonology, trauma and orthopedic surgery, rehabilitation, anesthesia and intensive therapy, neonatology, and newborn pathology with intensive care. In the hospital, there were on average 51.5 doctors (with the largest number of 72 doctors in 2011, and the least 45 doctors in 2017), and 191 nurses/midwives (with the largest number of 210 nurses in 2010, and the least 160 nurses in 2017). In 2017, a Hospital Emergency Department with 20 medical rescuers was opened. There were no ward maids employees. The hospital is cleaned by an outside company. Over the years 2010–2017, the number of patient-days in the hospital was systematically decreasing, as shown in Table 1.

### 3.2. Analysis of NSSI Occurring in the Hospital

In 2010–2017, 34 injuries were reported among medical personnel. Most incidents were in 2017-8, and the least in 2011-1. In 2014, no acute injuries were reported.

Most injuries were reported by nurses (*n* = 18). The most frequent injuries occurred when using a hollow-bore needle (*n* = 21). Most often, the staff injured themselves during surgery (*n* = 13) and injections (*n* = 9). There was only 1 incident of injury when recapping, at the beginning of the period under consideration (2010). The part of the body that was most vulnerable to injuries were fingers (*n* = 27). The most often injured were older workers over 40 (*n* = 26), on the morning shifts from 7 a.m.–1 p.m. (*n* = 18). Two times more NSSI were noted in the surgical wards than in non-surgical wards, but these differences were not significant. As a result of the injuries, PEP was implemented in 30 people. PEP was not carried out for 3 nurses and 1 ward maid. PEP was not implemented after a wounding with medical waste in the case of a ward maid in the medical room, and after a needle stick during blood collection and injections, in the delivery room and in the patients’ rooms, in the case of the nurses. (Table 2).

The average number of injuries per 100 nurses in the analyzed period was 1.22, and among physicians it was higher at 2.02/100 physicians. The overall injury rate per 100 hospital beds available ranged from 0.31 in 2011 to 2.52 in 2017 (average *IR_b_* = 1.34). The overall injury rate per 100,000 inpatient days ranged from 1.72 in 2011 to 19.60 in 2017 (average *IR_c_* = 8.59). With the exception of 2011, when no injuries were reported in nurses, *IR_c_* were higher in nurses than in physicians in all examined years. On average, *IR_c_* for nurses was 3.99 and for physicians 1.93, as shown in Table 3.

We also calculated *IR_c_* indicators, for 3-year periods before (2010–2012) and after (2015–2017) the entry into force of legal provisions requiring the reporting of NSSI. Both the rates of nurses, physicians, and general index for all staff members were higher after the entry of the relevant regulations than before their introduction (nurses: *IR_c_*_2010–2012_ = 4.23 vs. *IR_c_*_2015–2017_ = 5.82; physicians: *IR_c_*_2010–2012_ = 2.29 vs. *IR_c_*_2015–2017_ = 2.85; and all staff: *IR_c_*_2010–2012_ = 6.53 vs. *IR_c_*_2015–2017_ = 13.98).

## 4. Discussion

Occupational risk is understood as the probability of occurrence of undesired events related to the work performed. These events cause losses, among which the greatest role is played by the possibility of adverse health effects that may arise, because of occupational hazards occurring in the work environment. Occupational hazards for medical workers are caused by dangerous factors, among which biological, physical, chemical, and psychosocial factors are distinguished.

In a study from the Lodz region, it was shown that 81.9% of HCWs experience various types of situations threatening their safety [14]. Employees usually mentioned patients’ aggression, and only 13.7% of respondents noticed that a dangerous situation could be the transmission of microorganisms after NSSI. HCWs’ work is associated with continuous exposure to blood-borne infections. Occupational exposure to blood occurs among most medical workers. In the study conducted by Martins et al., 65% of employees at a selected hospital in Portugal reported having experienced at least one NSSI in the last 5 years [15]. Most of the events, similar to our study, and Gholami et al. [2] occurred among nurses. It should be noted that the percentage presentation of the frequency of injuries is not very accurate. Nurses represent the largest professional group in all hospitals around the world, which is why the percentages will be the highest among nurses. At the same time, the presented study draws attention to the fact that the number of personnel is decreasing year by year, therefore the lack of significant differences in the number of NSSI can be illusory. For this reason, the injury rates are better for comparison. In our study, the injury rates for every 100 working physicians, were two times higher than for nurses (average physicians 2.02/100 vs. nurses 1.22/100). Bush et al., however, showed higher rates of injuries for nurses (nurses 3.7/100 vs. physicians 1.6/100) [16]. These indicators allow the estimation of the number of injuries, for every 100-people exposed to injury. The indicator most often taken here is the number of staff employed. It should be remembered that this indicator will not consider holiday periods, sick times, part-time work, etc. Injury rate can be quantified considering the number of hospital beds. In our study, it amounted to 1.34/100 beds. However, this indicator also has its drawbacks. It considers all beds available in the hospital, but not necessarily all of them were occupied by patients throughout the entire period considered.

The third rate is an indicator that considers the number of inpatient days [13]. In our study, it amounted to an average of 3.99/100,000 for nurses and 1.93/100,000 for physicians and was higher than in another study (nurses 2.43/100,000 vs. physicians 0.23/100,000) [17]. This injury rate indicated that despite a small overall number of NSSI, and despite the decreasing number of inpatient days, injury rates increase, which may indicate an improvement in the reporting of such incidents. The calculation of the injury rates for 3-year periods prior to the introduction of legal provisions and after their implementation, also showed that legislation aimed at improving the registration of NSSI had the desired effect [18]. In the Ayranci and Kosgeroglu study, workers most often injured themselves during the injection (34.5%) [19]. In our study, the activity that carried the greatest chance of getting hurt was surgery. Similar to other studies, the most exposed parts of the body were the hands, and the majority of injuries occurred in the morning shifts [9,20,21,22]. In various studies, recapping needles was indicated as a frequent cause of injury [2,9,15]. We did not find such a problem in this hospital. The only such event took place at the beginning of the period under consideration.

PEP was implemented in most people after NSSI. Unfortunately, the records did not state the reason/argument for not implementing PEP, so it was difficult to find out why such a procedure was not carried out on the injured nurses or ward maid. The ward maid was injured with medical waste, so we assume that there was no known source of the needle or even a tool. After wounding with a tool, for example, a waste sack, one cannot determine which tool effected the injury. All the more so, without knowing the source to an injured person, it is absolutely necessary to implement PEP. In our case, the ward maids were employed by a different company than the hospital, so they must provide a safe working environment and PEP. The hospital may not have received feedback. Although in the remaining four cases of PEP lesions, PEP was implemented and the hospital knew about it. Thus, we noticed a lack of consistency in the information received and transmitted, and the need to harmonize the way of recording events. Many publications indicate underreporting [16,23,24]. The Regulation of the Minister of Health, regulates the rules for recording NSSI by imposing such an obligation [18]. In our study, the injury rates for the 3-year periods increased. However, it is possible, the data was still underestimated. For example, data showed that in 2014 (the year following the entering into force, of mandatory reporting of NSSI) no injuries were registered. Is it possible that absolutely nobody was hurt in the entire hospital? This seems unlikely. In our opinion, reporting will not be improved until the employees themselves understand the problem; until HCWs care about their own health. To raise HCWs awareness, systematic training should be a priority, but knowledge and awareness of threats are currently described with a fairly low level of risk [25]. This is evidenced by the fear associated with the treatment and care of HIV + patients, and frequent refusal of such assistance for infected patients [26]. Will the obligation to register change employers? In Poland, costs related to PEP are borne by the employer. Therefore, the employer “does not pay” to register applications. If the HCWs do not report NSSI, the employer does not know about the problem at all. Therefore, we believe that the initiative in the field of NSSI registration should come from HCWs, because it is their safety and health that is at risk, and health has a priceless value.

## 5. Conclusions

HCWs are routinely at risk of occupational exposure to blood and other potentially infectious materials. Most NSSIs are reported among nurses, but indicators based on injuries rates are more reliable for comparison than those based on frequency. Legislation on the need to register injuries seems to be necessary. In the supervision of the work safety of personnel, reliable reporting of all injuries by the concerned HCWs plays a key role.

## Figures and Tables

**Table 1:** Number of inpatients-days in selected departments by year.

Hospital Department	Years							
	2010	2011	2012	2013	2014	2015	2016	2017
Internal diseases	14,436	13,924	12,669	12,502	11,912	11,981	11,604	9692
Internal diseases with cardiological profile	14,126	14,001	13,383	12,908	11,875	11,640	11,297	9294
General surgery	6078	6906	6467	6505	6492	6633	6454	6203
Obstetrics with gynecology	12,267	11,350	11,804	11,542	10,849	9935	8181	6734
Pediatric surgery	3094	3309	3181	2713	2601	2413	2211	1801
Trauma and orthopedic surgery	5902	3596	3441	3500	3699	4089	4294	4050
Newborn pathology with intensive care	1440	1297	1178	1258	1003	919	808	428
Neonatology	3909	3491	809	4266	3829	4058	3128	2602
Total	61,252	57,874	52,932	55,194	52,260	51,668	47,977	40,804

**Table 2:** Injuries recorded by year depending on the variables studied (*n* = 34).

**Year**	**Profession**					**Medical Tool**				**Intervention**						**Location of Wounds**						**PEP**	
	**nurse**	**physician**	**practitioner**	**paramedic**	**Ward maid**	**Hollow-bore needle**	**Suture needle**	**scalpel**	**others**	**Blood collection**		**injection**	**surgery**	**recapping**	**cleaning**	**Abdominal cavity**	**Left finger**	**Left forearm**	**Left palm**	**Right finger**	**Right palm**	**yes**	**no**
2010	2	2	0	0	0	3	0	1	0	0		0	3	1	0	0	4	0	0	0	0	3	1
2011	0	1	0	0	0	0	0	1	0	0		0	1	0	0	0	0	0	1	0	0	1	0
2012	5	1	0	0	0	4	0	1	1	0		4	2	0	0	0	4	1	0	1	0	6	0
2013	1	0	0	2	1	4	0	0	0	0		3	0	0	1	0	3	0	0	0	1	4	0
2014	0	0	0	0	0	0	0	0	0	0		0	0	0	0	0	0	0	0	0	0	0	0
2015	2	1	0	0	1	2	0	2	0	1		0	2	0	1	0	1	0	0	1	2	3	1
2016	3	2	2	0	0	2	2	2	1	3		0	4	0	0	0	3	0	1	3	0	7	0
2017	3	1	1	0	3	6	1	0	1	2		2	1	0	3	1	4	0	0		0	6	2
Total	16	8	3	2	5	21	3	7	3	6		9	13	1	5	1	19	1	2	8	3	30	4
	Chi^2^ = 33.93, *p* = 0.086					Chi^2^ = 22.18, *p* = 0.57				Chi^2^ = 36.71, *p* = 0.047						Chi^2^ = 43.81, *p* = 0.05						Chi^2^ = 5.1, *p* = 0.531	
**Year**	**Place of Injury**						**Kind of Organizational Unit**							**Type of Department**		**Time of the Accident in a Day (Duty Hours)**			**Age of the Employee (Years)**				
	**Delivery room**	**dispensary**	**Operating suite**	**outside**	**Sick room**	**Treatment room**	**emergency**	**Internal**	**maternity**	**neonatal**	**surgical**		**others**	**surgical**	**Non-surgical**	**In the morning**	**In the afternoon**	**In the evening**	**20–30 y**	**31–40 y**	**41–50 y**	**51–60 y**	**>60 y**
2010	0	1	3	0	0	0	0	1	0	0	3		0	3	1	2	2	0	0	0	2	2	0
2011	1	0	0	0	0	0	0	0	1	0	0		0	1	0	1	0	0	0	0	0	1	0
2012	1	0	2	0	1	2	0	3	1	0	1		1	3	3	4	0	2	0	1	4	1	0
2013	1	0	0	0	3	0	2	1	1	0	0		0	3	1	1	2	1	1	0	0	3	0
2014	0	0	0	0	0	0	0	0	0	0	0		0	0	0	0	0	0	0	0	0	0	0
2015	0	0	2	1	1	0	0	1	1	0	1		1	2	1	1	2	1	0	1	2	1	0
2016	0	0	2	0	0	5	0	4	0	0	3		0	3	4	5	1	1	2	1	1	2	1
2017	2	0	1	2	2	1	2	1	0	2	2		1	6	1	4	1	3	2	0	2	4	0
Total	5	1	10	3	7	8	4	11	4	2	10		3	21	11	18	8	8	5	3	11	14	1
	Chi^2^ = 45.64, *p* = 0.034						Chi^2^ = 37.09, *p* = 0.174							Chi^2^ = 9.10, *p* = 0.694		Chi^2^ = 10.52, *p* = 0.571			Chi^2^ = 20.17, *p* = 0.687				

Chi^2^—Pearson chi^2^ test; *p*—significance level; PEP—post-exposure prophylaxis.

**Table 3:** Injury rates *IR_p_*, *IR_b_*, *IR_c_* by years among HCWs.

Year	*IR_p_*/100			*IR_b_*/100	*IR_c_*/100,000		
	Injury Rate among Nurses	Injury Rate among Physicians	Injury Rate among Nurses and Physicians	Injury Rate among All Personnel	Injury Rate among Nurses	Injury Rate among Physicians	Injury Rate among All Personnel
2010	0.95	2.99	1.44	1.26	3.26	3.26	6.53
2011	0.00	1.39	0.36	0.31	0.00	1.72	1.72
2012	2.42	1.49	2.19	1.89	9.44	1.88	11.33
2013	0.54	0.00	0.40	1.26	1.81	0.00	7.24
2014	0.00	0.00	0.00	0.00	0.00	0.00	0.00
2015	1.05	2.00	1.24	1.26	3.87	1.93	7.74
2016	1.70	4.08	2.22	2.20	6.25	4.16	14.59
2017	1.88	2.22	1.95	2.52	7.35	2.45	19.60
average	1.22	2.02	1.40	1.34	3.99	1.93	8.59

*IR_p_*—Injury rate depending on the profession; *IR_b_*—Injury rate depending on the number of hospital beds; and *IR_c_*—Injury rate depending on the number of inpatient-days.

## References

[1] Pruss-Ustun A., Rapiti E., Hutin Y. (2003). Sharps Injuries: Global Burden of Disease from Sharps Injuries to Health-Care Workers.

[2] Gholami A., Borji A., Lotfabadi P., Asghari A. (2013). Risk factors of needlestick and sharps injuries among healthcare workers. Int. J. Hosp. Res..

[3] Centers for Disease Control and Prevention (1989). Guidelines for prevention of transmission of human immunodeficiency virus and hepatitis B virus to health-care and public-safety workers. MMWR Morb. Mortal. Wkly. Rep..

[4] Centers for Disease Control and Prevention (1998). Recommendations for prevention and control of hepatitis C virus (HCV) infection and HCV-related chronic disease. MMWR Morb. Mortal. Wkly. Rep..

[5] Pittet D., Donaldson L. (2005). Clean care is safer care: The first global challenge of the WHO World Alliance for Patient Safety. Infect. Control Hosp. Epidemiol..

[6] Pittet D., Donaldson L. (2006). Challenging the world: Patient safety and health care-associated infection. Int. J. Qual. Health Care.

[7] Świątkowska B., Hanke W., Szeszenia-Dąbrowska N. (2017). Occupational Diseases in Poland in 2016.

[8] Rybacki M., Piekarska A., Wiszniewska M., Walusiak-Skorupa J. (2013). Work safety among polish health care workers in respect of exposure to bloodborne pathogens. Med. Pr..

[9] Jahangiri M., Rostamabadi A., Hoboubi N., Tadayon N., Soleimani A. (2016). Needle stick injuries and their related safety measures among nurses in a University Hospital, Shiraz, Iran. Saf. Health Work.

[10] Ganczak M., Milona M., Szych Z. (2006). Nurses and occupational exposures to bloodborne viruses in Poland. Infect. Control Hosp. Epidemiol..

[11] Ganczak M., Szych Z., Karakiewicz B. (2012). Assessment of occupational exposure to HBV, HCV and HIV in gynecologic and obstetric staff. Med. Pracy.

[12] Nagandla K., Kumar K., Bhardwaj A., Muthalagan D., Yhmin C., Lun L.W., Shi W.W., Razak N.I. (2015). Prevalence of needle stick injuries and their underreporting among healthcare workers in the department of obstetrics and gynaecology. Int. Arch. Med..

[13] Beaglehole R., Bonita R., Kjellstrom T. (1993). Basic Epidemiology.

[14] Garus-Pakowska A., Szatko F. (2011). Percutaneous exposures in medical personnel. Med. Pr..

[15] Martins A., Coelho A.C., Vieira M., Matos M., Pinto M.L. (2012). Age and years in practice as factors associated with needlestick and sharps injuries among health care workers in a Portuguese hospital. Accid. Anal. Prev..

[16] Bush C., Schmid K., Rupp M.E., Watanabe-Galoway S., Wolford B., Sandkovsky U. (2017). Bloodborne pathogen exposures: Difference in reporting rates and individual predictors among health care personnel. Am. J. Infect. Control.

[17] Motaarefi H., Mahmoudi H., Mohammadi E., Hasanpour-Dehkordi A. (2016). Factors associated with needlestick injuries in health care occupations: A systematic review. J. Clin. Diagn. Res..

[18] Regulation of the Minister of Health of 6 June 2013 on Occupational Safety and Health in the Performance of Work Related to the Severity of Injuries Caused by Acute Use of Health Services. http://isap.sejm.gov.pl/DetailsServlet?id=WDU20130000696.

[19] Ayranci U., Kosgeroglu N. (2004). Needlestick and sharps injuries among nurses in the healthcare sector in a city of western Turkey. J. Hosp. Infect..

[20] Parsa-Pili J., Izadi N., Golbabaei F. (2013). Factors associated with needle stick and sharp injuries among Health Care Workers. Int. J. Occup. Hyg..

[21] Bhardwaj A., Sivapathasundaram N., Yusof M., Minghat A., Swe K., Sinha N. (2014). The prevalence of accidental needle stick injury and their reporting among Healthcare Workers in Orthopaedic Wards in General Hospital Melaka, Malaysia. Malays. Orthop. J..

[22] Pasha L., Farid H., Faisal M.R. (2016). Dental Professionals Experience Regarding Sharp Injuries during Practice. Pak. Oral Dent. J..

[23] Winchester S.A., Tomkins S., Cliffe S., Batty L., Ncube F., Zuckerman M. (2012). Healthcare workers’ perceptions of occupational exposure to blood-borne viruses and reporting barriers: A questionnaire-based study. J. Hosp. Infect..

[24] Lazenby M.G., Anderud J., Whitley S.P. (2011). Blood-borne viruses: Are we taking them seriously? A survey of UK oral and maxillofacial surgeons. Br. J. Oral Maxillofac. Surg..

[25] Shaghaghian S., Pardis S., Mansoori Z. (2014). Knowledge, attitude and practice of dentists towards prophylaxis after exposure to blood and body fluids. Int. J. Occup. Environ. Med..

[26] Garus-Pakowska A., Górajski M., Szatko F. (2017). Knowledge and attitudes of dentists with respect to the risks of blood-borne pathogens—A cross-sectional study in Poland. Int. J. Environ. Res. Public Health.

